# Supplementary material for: An andesitic source for Jack Hills zircon supports onset of plate tectonics in the Hadean
Source: Nat Commun. 2020 Mar 6;11:1241. doi: 10.1038/s41467-020-14857-1 (PMC7060172; doi:10.1038/s41467-020-14857-1)

Supplementary Figure 1: Plot of Th/Y versus SiO2 (wt. %), with dots and dashed curves showing the maximum, minimum and geometry mean of the data (see Methods section for full explanation).

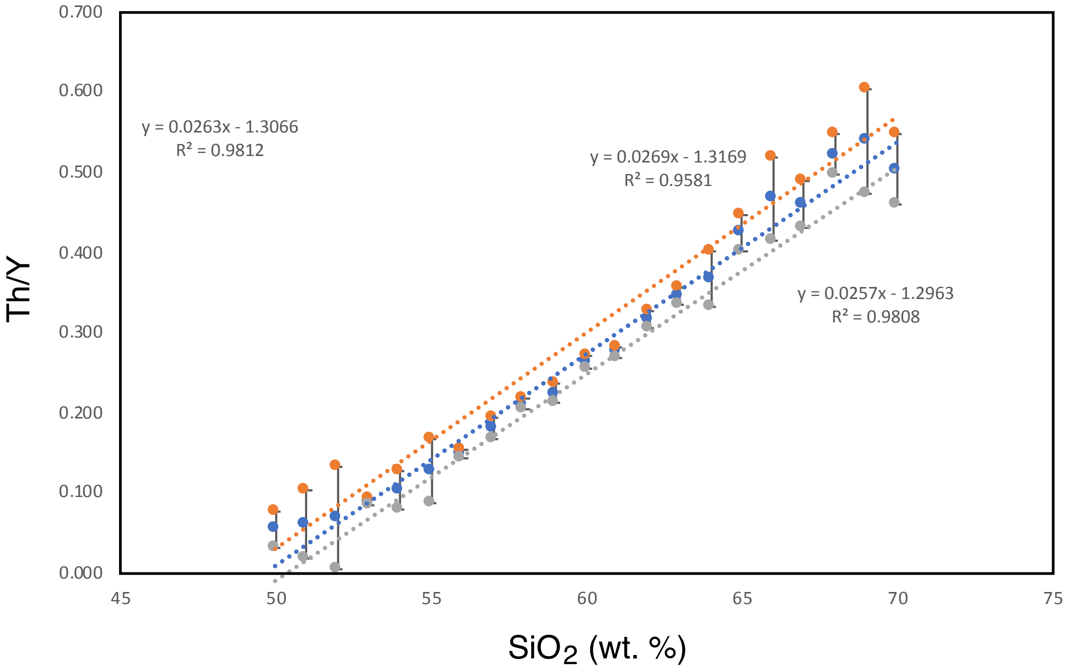

Supplement: Supplementary file 2 — Supplementary Information [file 41467_2020_14857_MOESM2_ESM.pdf]
