# Supplementary material for: An andesitic source for Jack Hills zircon supports onset of plate tectonics in the Hadean
Source: Nat Commun. 2020 Mar 6;11:1241. doi: 10.1038/s41467-020-14857-1 (PMC7060172; doi:10.1038/s41467-020-14857-1)
Supplement: Supplementary file 3 — Description of Additional Supplementary Files [file 41467_2020_14857_MOESM3_ESM.pdf]

## Description of Additional Supplementary Files

File Name: Supplementary Data 1

Description: SHRIMP U/Pb isotope data

File Name: Supplementary Data 2

Description: Zircon trace element data
